# The Paramount Role of *Drosophila melanogaster* in the Study of Epigenetics: From Simple Phenotypes to Molecular Dissection and Higher-Order Genome Organization

**DOI:** 10.3390/insects12100884

**Published:** 2021-09-29

**Authors:** Jean-Michel Gibert, Frédérique Peronnet

**Affiliations:** Centre National de la Recherche Scientifique (CNRS), Laboratoire de Biologie du Développement (LBD), Institut de Biologie Paris Seine (IBPS), Sorbonne Université, 75005 Paris, France

**Keywords:** *Drosophila*, epigenetics, chromatin, heterochromatin, piRNAs, polycomb, trithorax, histones, higher-order chromatin structure, environment

## Abstract

**Simple Summary:**

Since its adoption as a model organism more than a hundred years ago, the fruit fly *Drosophila melanogaster* has led to major discoveries in biology, notably in epigenetics. Epigenetics studies the changes in gene function inherited through mitosis or meiosis that are not due to modifications in the DNA sequence. The first discoveries in epigenetics emerged from analyses of the perturbations of simple phenotypes such as the bristle position or cuticle pigmentation. Identification of the mutated genes led to the discovery of major chromatin regulators, which were found to be conserved in other insects, and unexpectedly, in all metazoans. Many of them deposit post-translational modifications on histones, the proteins around which the DNA is wrapped. Others are chromatin remodeling complexes that move, eject, or exchange nucleosomes. We review here the role of *D. melanogaster* research in three important epigenetic fields: The formation of heterochromatin, the repression of mobile DNA elements by small RNAs, and the regulation of gene expression by the antagonistic Polycomb and Trithorax complexes. We then review how genetic tools available in *D. melanogaster* have allowed us to examine the role of histone marks and led to more global discoveries on chromatin organization. Lastly, we discuss the impact of varying environmental conditions on epigenetic regulation.

**Abstract:**

*Drosophila melanogaster* has played a paramount role in epigenetics, the study of changes in gene function inherited through mitosis or meiosis that are not due to changes in the DNA sequence. By analyzing simple phenotypes, such as the bristle position or cuticle pigmentation, as read-outs of regulatory processes, the identification of mutated genes led to the discovery of major chromatin regulators. These are often conserved in distantly related organisms such as vertebrates or even plants. Many of them deposit, recognize, or erase post-translational modifications on histones (histone marks). Others are members of chromatin remodeling complexes that move, eject, or exchange nucleosomes. We review the role of *D*. *melanogaster* research in three epigenetic fields: Heterochromatin formation and maintenance, the repression of transposable elements by piRNAs, and the regulation of gene expression by the antagonistic Polycomb and Trithorax complexes. We then describe how genetic tools available in *D. melanogaster* allowed to examine the role of histone marks and show that some histone marks are dispensable for gene regulation, whereas others play essential roles. Next, we describe how *D. melanogaster* has been particularly important in defining chromatin types, higher-order chromatin structures, and their dynamic changes during development. Lastly, we discuss the role of epigenetics in a changing environment.

## 1. Introduction

Since its adoption as a genetic model by Thomas H. Morgan more than a hundred years ago, *Drosophila melanogaster* has become one of the most studied organisms. It has allowed major discoveries in most fields of biology, which notably led to the attribution of Nobel prizes to several *Drosophila* geneticists. In particular, *Drosophila* has proven invaluable for the study of epigenetic mechanisms. Epigenetics, initially defined as a bridge between the phenotype and the genotype [1], is nowadays described as the study of mitotically and/or meiotically heritable changes in gene function that cannot be explained by changes in DNA sequence [2]. Epigenetic processes were uncovered in *Drosophila* as a result of studies of simple phenotypes (eye or cuticle pigmentation, appendage morphology, position of bristles, organization of larval denticles). Then, the development of numerous genetic tools made it possible to finely dissect the systems and led to the identification of genes and regulatory sequences at play. The cloning of some of these genes allowed the production of antibodies and localization of the corresponding proteins on chromatin, first on salivary gland polytene chromosomes and later on the whole genome by Chromatin Immunoprecipitation (ChIP) (ChIP-on-chip and then ChIP-seq) thanks to the sequencing of *Drosophila* genome in 2000 [3]. Major chromatin regulator complexes were then purified. Many of them contain histone modifying enzymes that add the so-called epigenetic marks. These complexes are widely conserved among animals, and some even in yeasts or plants [4]. The subcellular localization of these chromatin regulators and more recent techniques such as chromosome conformation captures (3C) have led to the identification of nuclear territories and a higher-order chromatin organization [5]. Genetic tools developed in *D. melanogaster* have made it possible to follow territories during development, and to demonstrate that they are very dynamic.

Among the different epigenetic processes discovered in *D. melanogaster,* three stand out for they are remarkably conserved: The formation and maintenance of heterochromatin, the regulation of transposons by piRNA clusters, and the maintenance of gene expression by the Polycomb and Trithorax complexes. We will first briefly review these three mechanisms, then describe a few innovative studies developed in *D. melanogaster* that have been fundamental for the understanding of epigenetics, and conclude, through several examples, by briefly addressing what is of growing interest in light of climate change, namely the impact of the environment on genome expression and epigenetic mechanisms.

## 2. Formation and Maintenance of Heterochromatin

**Figure 1:**
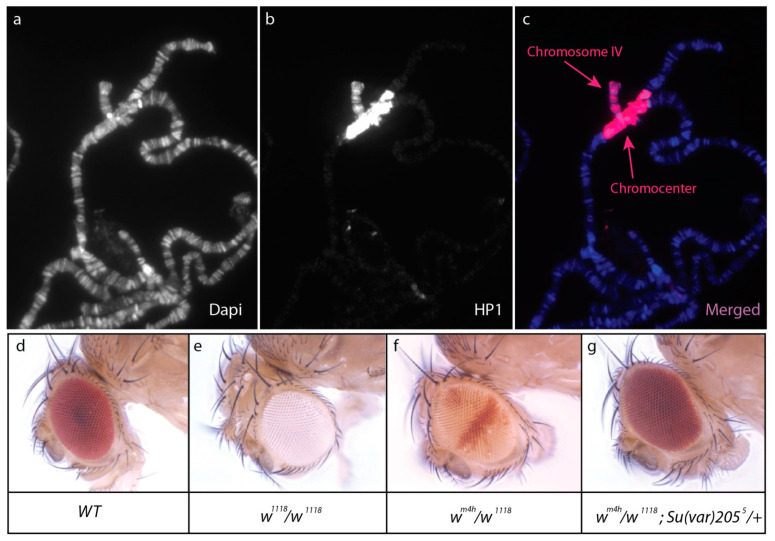
Heterochromatin represses neighboring genes. (**a**–**c**): HP1a immunostaining of salivary gland polytene chromosomes reveals the heterochromatic nature of chromosome IV and the chromocenter. DNA is stained with DAPI (in blue). (**d**–**g**): Position effect variegation of *white* (*w*) and the effect of a mutation in *Su(var)205*. Wild-type flies have red eyes (**d**), whereas flies carrying a loss of function mutation of *w* (*w*^1118^) have white eyes (**e**). The *w^m^*^4*h*^ inversion relocates *w* close to the pericentromeric heterochromatin; silencing of *w* by the stochastic spreading of heterochromatin leads to a mosaic phenotype (**f**). Mutation in *Su(var)205* encoding HP1a dominantly suppresses this variegation (**g**).

Heterochromatin is a highly compacted type of chromatin mainly located in centromeres and telomeres of chromosomes and marked by specific proteins such as HP1 (Heterochromatin Protein 1), a histone H3 variant called CENP-A in *D. melanogaster*, and an epigenetic mark that is di- or tri-methylation of H3 on lysine 9 (H3K9me2/3). The heterochromatin contains few genes, many repeated sequences, and many transposons [6]. Chromosomal rearrangements (translocation, inversion), which consequently relocate marker genes (*white*, *yellow*, *Stubble,* etc.) close to the heterochromatic centromere and its surrounding region called pericentromeric heterochromatin, have been invaluable tools to identify genetic factors involved in the maintenance of heterochromatin (reviewed in [7,8]). Indeed, the absence of insulator regions leads to unequal heterochromatin spreading onto the marker gene depending on its distance from heterochromatin. This causes stochastic silencing of the marker gene, a phenomenon called Position Effect Variegation (PEV), which translates into a mosaic phenotype. PEV is also observed with transgenes inserted in other heterochromatic regions such as the Y or the fourth chromosome, which are almost entirely heterochromatic in *D. melanogaster* [9]. PEV assays allowed for screening of Enhancers or Suppressors of variegation (*E(var)* and *Su(var)*) that prevent the formation or maintain heterochromatin, respectively [10] (Figure 1d–g).

HP1, one of the major components of heterochromatin, was identified in *D. melanogaster* by screening a phage expression library with antibodies designed against unknown chromosomal proteins extracted from nuclei [11]. It was later shown that HP1 (now called HP1a) was encoded by *Su(var)205* [12]. Immunostaining of HP1a on polytene chromosomes labels telomeres, the pericentromeric regions, the Y, and the fourth chromosome [13] (Figure 1a–c). In addition, HP1a represses certain genes outside the centromere, for example, it is recruited by Eyegone for the repression of *wingless* in the eye imaginal disc [14]. However, HP1a is also present on several expressed genes and is required for their expression [15,16]. *HP1a* belongs to a highly conserved multigenic family, which evolved rapidly in the *Drosophila* genus [17]. *HP1* paralogues are expressed in different tissues where they play distinct roles [18]. For example, *Rhino* (alias *HP1d*) and *HP1e* are expressed in ovaries and testes, respectively. *Rhino* plays a major role in silencing transposable elements in the female germline (see below).

*Su(var)3-9* encodes a highly conserved methyltransferase that trimethylates lysine 9 of histone 3 (H3K9me2/3), the epigenetic mark recognized by the chromodomain of HP1a. Two other methyltransferases of H3K9 were identified in *D. melanogaster* by conservation with their vertebrate homologues, SetdB1/eggless and G9a [19]. Immunostaining of polytene chromosomes showed that their roles are partially distinct. Whereas *Su(var)3-9* is required for H3K9 di/tri methylation and HP1a localization in pericentromeric regions [20], *SetdB1/eggless* is required for H3K9 methylation and HP1a binding on the fourth chromosome [21,22]. More precise ChIP-on-chip studies confirmed these results but showed that some small regions of this chromosome also require *Su(var)3-9* for HP1a binding [23]. Interestingly, the enhancer of variegation *JIL-1* encodes a kinase of H3S10 [24]. Loss-of-function mutations for *JIL-1* indeed lead to ectopic spreading of HP1 on chromosomal arms [25]. This effect decreases when the dose of *Su(var)3-9* is reduced, showing that there is a balance between Su(var)3-9 and JIL-1 and their respective epigenetic marks H3K9me2/3 and H3S10p for the maintenance of pericentromeric heterochromatin [26].

Recent studies have extended the list of heterochromatin components and have provided new insights into its formation. For example, a study associating the purification of HP1a interactors and a genome-wide RNAi screen showed that HP1a interacts with many other chromatin proteins and unexpectedly revealed that heterochromatin forms dynamic sub-domains during cell division [27]. Another recent study showed that the formation of heterochromatin is mediated by liquid phase separation [28]. Small HP1 foci form via nucleation of multiple HP1 molecules and other heterochromatin components via weak hydrophobic interactions then fuse to form larger droplets. These results do not invalidate the importance of physical interactions between HP1 and its interactors in the formation of heterochromatin, but dramatically change our view on this nuclear membrane-free compartment. They could explain the association between a distal heterochromatic domain and the main domain made of centromeric and pericentromeric heterochromatin that was reported [29].

## 3. Regulation of Transposons by piRNA Clusters

The piRNA clusters are heterochromatic loci containing fragments of transposable elements that protect the genome against the deleterious influence of these mobile genes [30]. Indeed, they produce small non-coding RNAs 23 to 30 nucleotides long, which, by complementarity, drive the slicing of transposable element transcripts by interacting with PIWI proteins (hence the name piRNA), thus ensuring post-transcriptional silencing of these transposons [31,32]. piRNA clusters were first discovered in *Drosophila* as they repress transposons in the ovaries. Notably, *flamenco,* which contains fragments of *gypsy* and *ZAM* retrotransposons, was among the first piRNA clusters discovered (for review [33]). However, at that time, the molecular nature of *flamenco* was unknown and its localization at the boundary between euchromatin and heterochromatin on the X chromosome in a region full of transposon remnants made its characterization difficult. It was only in 2007 that *flamenco* was identified not as a protein coding gene, but as one of the piRNA producing loci [30]. *flamenco* is actively transcribed in ovarian somatic cells and its transcription requires trimethylation of H3K9 by Egless/SetdB1 and the transcription factor Cubitus Interruptus [34,35]. Like other piRNA clusters active in somatic cells, *flamenco* is a uni-strand cluster. Indeed, there are two kinds of piRNA clusters in the ovary. In somatic cells, piRNA clusters are uni-strand, that is, their transcription proceeds in only one direction and does not require the HP1 homologue Rhino. Furthermore, their transcripts are spliced [35]. In contrast, in germinal cells, the piRNA clusters are mainly dual-strand (i.e., transcription proceeds in both directions), transcripts are not spliced and their transcription requires Rhino [36,37]. In these cells, PIWI and transgenerationally inherited piRNAs are required for the deposition of H3K9me3 on dual-strand clusters [38,39]. Rhino, which forms a complex with Deadlock and Cutoff associates with H3K9me3 [37,40]. Deadlock, by interacting with the transcription factor Moonshiner, allows the recruitment of the core transcriptional machinery [41] (for a review see [42]). In a second step, Eggless/SetdB1 is recruited to targeted transposons by piRNA silencing complexes via the factor Silencio [43]. The tri-methylation of H3K9 by Eggless/SetdB1 allows the recruitment of HP1a and, in parallel, PIWI recruits the linker histone H1, which leads to heterochromatinization of targeted transposons and reinforces transcriptional silencing [44]. Hence, piRNAs target transposons transcriptionally and post-transcriptionally.

**Figure 2:**
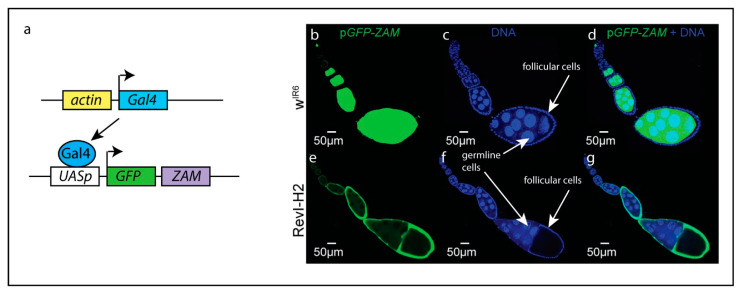
Regulation of the retrotransposon *ZAM* by piRNAs. (**a**): Principle of the *ZAM* sensor. Expression of the trans-activator Gal4 is ubiquitously driven by *actin* regulatory sequences. The GFP coding sequence is fused to a fragment of the *ZAM* *env* region and placed under the control of UAS sequences and a minimal promoter. (**b**–**g**): Expression of the ZAM sensor in ovaries of control flies (*w^IR^*^6^) or in flies with a deletion in *flamenco* and a *de novo* insertion of *ZAM* in a pre-existing dual strand cluster (*RevI-H2*) (**b**,**d**,**e**,**g**). In *w^IR^*^6^, the sensor is silenced by the uni-strand piRNA cluster *flamenco* in somatic follicular cells and expressed in the germline (**b**,**d**). On the opposite, in *RevI-H2*, the sensor is expressed in the somatic follicular cells due to the mutation in *flamenco* and silenced in the germline due to the *ZAM* insertion in the dual strand cluster (**e**,**g**). Origin of the photographs: Courtesy of Emilie Brasset [45] (http://creativecommons.org/licenses/by/4.0/ accessed 2 September 2021). In blue: Staining of DNA with DAPI. In green: GFP-ZAM.

A recent study used small RNA sequencing and a transgenic fly expressing a sensor for the retrotransposon *ZAM* (Figure 2a) to analyze the production of *ZAM* piRNAs in ovaries [45]. As expected, in the control strain, the *ZAM* sensor was repressed by *flamenco* in somatic cells while highly expressed in germ line cells (Figure 2b–d). In a *flamenco* mutant where the region containing *ZAM* fragments was deleted, it was expected that the *ZAM* sensor would be expressed in both somatic and germline cells as no *ZAM* piRNAs were produced. However, the sensor was expressed only in somatic cells (Figure 2e–g). Accordingly, the production of *ZAM* piRNAs was strongly increased in ovaries, which resulted from a new insertion of *ZAM* in a dual-strand piRNA cluster active in germline cells. In the *flamenco* mutant, the derepressed *ZAM* retrotransposons probably invaded the neighboring germline leading to a new insertion in a piRNA cluster that therefore protected the germline against deleterious insertions. This new insertion was probably favored by natural selection, which maintained it in the population. These observations provide clues about the mechanisms by which piRNA clusters evolve.

piRNA clusters can exist in two distinct states, either inactive or active, depending on whether or not they produce piRNAs. This was demonstrated by studying an artificial piRNA cluster made of P element-derived transgenes. The piRNAs produced by the active cluster silenced P elements located elsewhere in the genome [46]. The inactive or active state of this piRNA cluster can be stably maintained across generations, making it the first case of transgenerational epigenetics reported in *Drosophila*. Remarkably, maternal inheritance of piRNAs can convert a paternal inactive piRNA cluster into an active one, an epigenetic conversion called paramutation [37,46]. The newly activated piRNA cluster is then maintained across generations and becomes paramutagenic itself.

## 4. Polycomb and Trithorax Complexes and the Maintenance of Chromatin Conformation

The *Polycomb-group* (*PcG*) and *Trithorax-group* (*TrxG*) genes were first identified in *D. melanogaster* as regulators of homeotic (*Hox*) genes [47,48]. *PcG* genes encode proteins that maintain the repression of *Hox* genes after the initial specification of their expression pattern whereas *TrxG* genes were initially genetically identified as activators of *Hox* genes and antagonists of *PcG* [49].

**Figure 3:**
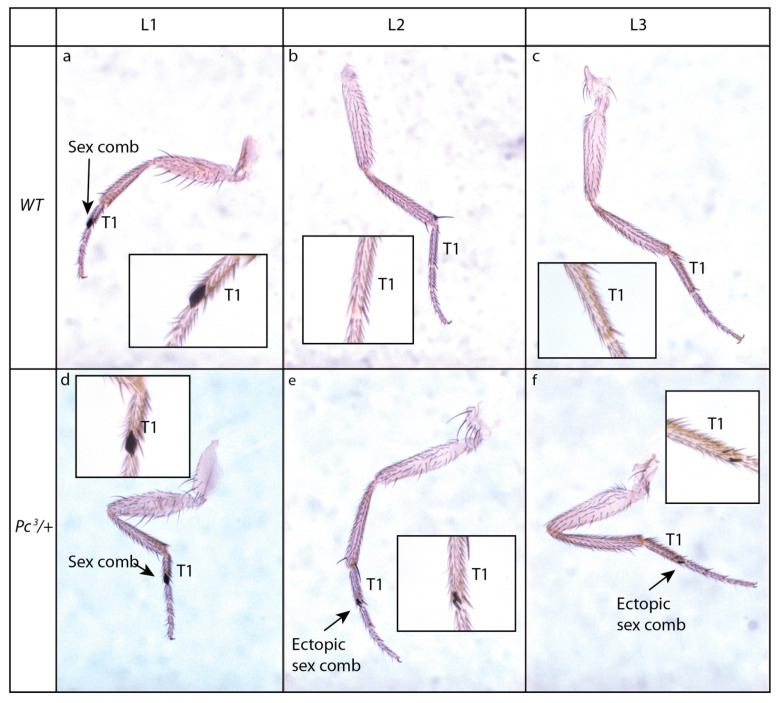
Ectopic sex combs of a *PcG* mutant. (**a**–**f**): Sex comb phenotype of *Polycomb* mutant. Sex combs are organs made of modified bristles present in males on the first tarsal segment (T1) of the first legs (L1) (**a**–**c**). The Polycomb mutant *Pc*^3^ shows dominantly ectopic partial sex combs on more posterior legs (L2, L3) (**d**–**f**).

*PcG* mutations induce ectopic expression of *Hox* genes and a change of segment identity leading to homeotic phenotypes. The name of many *PcG* genes relates to these homeotic phenotypes (i.e., *polyhomeotic*, *pleiohomeotic*) or to the presence of ectopic sex combs. Sex combs are organs made of modified bristles normally present on the first tarsal segments of the most anterior pair of legs in males (Figure 3a–c). Ectopic sex combs are frequently observed in *Drosophila* males that are mutant for *PcG* (i.e., *Polycomb*, *Polycomb-like*, *Posterior sex combs*, *extra sexcombs*, *Sex combs extra*, *super sex combs*, *multi sex combs, etc.*) (Figure 3d–f). This is caused by ectopic expression of the *Hox* gene *Sex combs reduced* [50]. Sequencing of *Polycomb* revealed the existence of a chromatin-addressing domain shared with HP1 [51], named chromodomain (for chromatin organization modifier), and Polycomb (PC) was further shown to be involved in chromatin packaging [52].

Most PcG proteins are part of large chromatin binding complexes e.g., Polycomb Repressive Complex 1 (PRC1), Polycomb Repressive Complex 2 (PRC2), or Polycomb Repressive Deubiquitinase (PR-DUB) [53,54,55,56,57,58,59]. Several of them have histone-modifying activities. For example, the PRC1 complex contains the enzyme dRing (encoded by *Sex combs extra*) that ubiquitinates H2AK118; PR-DUB contains Calypso, the enzyme that removes this ubiquitin residue; PRC2 contains the enzyme E(Z) that tri-methylates H3K27 (H3K27me3), etc. [54,58,59,60,61]. This repressive mark is first established early in the *Drosophila* embryo by maternal E(Z), and prevents precocious activation of lineage-specific genes at zygotic genome activation [62]. Furthermore, core components of PRC1 were shown to compact nucleosomal arrays in vitro [63]. Other *PcG* genes encode proteins with different molecular activities. For example, *multi sex combs* and *cramped* encode regulators of histone gene expression [64,65,66,67], *super sex combs* encode a glycosyltransferase of Polyhomeotic, a PRC1 member, and is essential for PRC1 function [68].

*TrxG* genes are required for the maintenance of *Hox* gene activation after the initiation of their expression. Consequently, loss-of-function alleles of *TrxG* genes lead to a loss of *Hox* gene expression and homeotic phenotypes [48]. For example, a mutant for *trithorax* (*trx*) presents a partial transformation of the halters (modified wings located on the third thoracic segment in Diptera) into wings caused by a decrease in the *Hox* gene *Ultrabithorax* (*Ubx*) expression. Similar to *PcG* genes, *TrxG* genes are widely conserved and their products form complexes of which some members encode histone-modifying enzymes. Several TrxG complexes harbor histone methyl-transferase activity, for example, the TAC1 complex (Trithorax Activating Complex 1) methylates H3K4, the family of COMPASS complexes (SET1, Trithorax dCOMPASS-like, and Trithorax-related dCOMPASS-like) also methylates H3K4, the AMC complex methylates H3K36, and the DotCom complex methylates H3K79 [69,70,71,72]. TAC1 also displays histone acetylase activity, which targets several lysines of histones H3 and H4 [73]. The second group of TrxG complexes displays chromatin remodeling activity due to an ATPase sub-unit such as the BAP (Brahma-Associated Protein) and the PBAP (Polybromo-containing BAP) complexes [74]. As for PcG, and as revealed by genome-wide studies, the regulatory role of TrxG goes far beyond *Hox* genes, and the deposited histone marks are widely observed across the epigenome [75]. Remarkably, the antagonism between *PcG* and *TrxG* genes discovered years ago by the first genetic experiments in *D. melanogaster* was confirmed later by studying the enzymatic activities of the complexes. Indeed, some of the histone marks deposited by TrxG complexes directly antagonize PcG ones, for example, H3K36 methylation and H3K27 acetylation deposited by AMC and TAC1, respectively, prevent H3K27 methylation by PRC2 [76,77]. Conversely, Polycomb interacts with CBP and reduces H3K27 acetylation by TAC1 [78]. Furthermore, PRC1 inhibits chromatin remodeling by the BRM complex [79].

Genome-wide analyses in *D. melanogaster* have shown that PcG complexes, as well as TrxG ones, regulate many more genes than *Hox* [75]. Indeed, several hundred genes are bound by these complexes and many of them encode developmental regulators. The crucial issue of their recruitment was addressed in *D. melanogaster* by taking advantage of the numerous genetic tools available. It was found that, on the one hand, complexes’ recruitment depends on the sequence of each target and many DNA binding factors with sequence specificity, such as Pleiohomeotic (PHO), the GAGA factor (GAF), Pipsqueak (Psq), Grainyhead (Grh), Dorsal switch protein 1 (Dsp1) or Zeste (Z), participate [53,80,81,82,83,84]. On the other hand, PcG and TrxG complexes bind to promoters and gene bodies where they interfere with transcriptional initiation or elongation [75,85]. Bioinformatics and functional studies also revealed the existence of Polycomb and Trithorax Response Elements (now named PREs) in the *cis*-regulatory sequences of the PcG/TrxG target genes. Strikingly, the presence of a PRE in a transgene was sufficient to induce the formation of a new binding site for PcG proteins on the polytene chromosome [55,56]. An analysis of transgenic lines carrying a PRE juxtaposed to reporter genes showed that it has the ability to induce PcG-dependent silencing of the reporter [86]. Furthermore, using an inducible system, it was possible to demonstrate that activation of the reporter during a short period of embryonic life revealed PRE-dependent maintenance of activation during development [87]. PREs were thus demonstrated to be central for epigenetic transmission of transcriptional states.

Another crucial issue was the persistence of chromatin conformation and its associated epigenetic states through chromatin replication. The first indication that complexes could perpetuate the mark themselves occurred when it was shown that the PRC2 complex not only writes the H3K27me3 epigenetic mark but also binds to it, suggesting that it could mark newly incorporated histones [88]. More recently, two important studies have proven that maintenance of the chromatin state during DNA replication implicates TrxG and PcG proteins themselves [89,90]. Notably, TRX and E(Z) remain associated with the newly replicated DNA whereas histone H3 trimethylated on lysines 4 or 27 are replaced by non-methylated H3 after DNA replication. The epigenetic marks would then be re-established after the S-phase of the cell cycle. The importance of PRC2 to propagate H3K27me3 to newly incorporated histones during replication was confirmed in recent studies using transgenes containing a *Hox* gene PRE. PRC2 is recruited on the PRE and H3K27me3 propagates on the flanking regions. Excision of the PRE leads to dilution of H3K27me3 at each DNA replication cycle, showing that the newly written epigenetic mark is not sufficient to recruit PRC2 and to maintain itself in the long term [91,92].

## 5. Testing the Role of Histone Marks in *Drosophila*

The combination of histone marks present on a gene correlates with its transcriptional status, which led to the notion of the “histone code” formulated twenty years ago, which implied that such combinations would recruit specific chromatin-binding proteins, thus driving the levels and duration of gene expression [93]. Elegant studies have been performed to test the role of individual histone marks by mutating histone genes [94,95,96,97]. In *D. melanogaster*, canonical histones are encoded by a large complex (*HisC*) formed by 23 repeats of a 5 kb unit containing one of each histone gene (*H1*, *H2A*, *H2B*, *H3.2,* and *H4*). Histones also display variants encoded by genes scattered in the genome that have more specific functions. For example, the presence of the H3.3 variant of H3 correlates with sites of active transcription. To test the role of given residues, deficiencies covering the histone cluster were rescued with transgenes containing between 6 and 12 repeats of wild-type or mutated histone genes. Strikingly, it was shown that H3.2 and H3.3 can compensate each other, provided that their timing of transcription was respected [94]. To address the role of H3K4me3, supposedly critical for gene activation, lysine 4 of canonical H3.2 and variant H3.3 were mutated into alanine or arginine. Unexpectedly, these mutations did not affect the expression of most of the genes analyzed, even if some of them, for example *Ubx*, were slightly less expressed [94]. The authors made the hypothesis that H3K4me3 might contribute to robust transcription under stress but not in standard environmental conditions. Similarly, methylation of H3K36, believed to be involved in transcriptional elongation, might in fact not be essential for the regulation of gene expression. Indeed, *Hox* genes, which are very sensitive targets of Ash1, the H3K36 methylase of the AMC complex, are not greatly repressed in embryos where H3K36 is mutated to H3R36 in both canonical and variant proteins [96]. In contrast, mutation of H3K9 into H3R9 results in a decrease in chromatin compaction accompanied by deregulation of piRNA clusters and transposons, thus inducing their mobilization [97]. In imaginal discs, clones of H3K27 to H3R27 mutant cells ectopically express *Hox* genes, similar to *PcG* gene mutant cells, showing that H3K27me3 is essential for Polycomb silencing [95]. Hence, these experiments have allowed researchers to precisely address the role of histone modifications in the control of gene expression.

## 6. Towards an Integrated Vision of Chromatin Domains

Genome-wide approaches analyzing the binding patterns of several chromatin proteins or histone marks in different cell lines or tissues have provided a more global description of chromatin types [98,99]. A pioneering study followed the binding sites of 53 non-histone chromatin proteins in the *Drosophila* Kc cell line using bacterial DNA methyltransferase (DamID) [98]. This allowed the identification of five main types of chromatin described with a color code: (i) Green chromatin or heterochromatin, silenced and marked notably by HP1 and H3K9me2; (ii) blue chromatin or PcG chromatin, also silenced but enriched in H3K27me3; (iii) red chromatin, in which genes are expressed, is rich in RNA polII, the TrxG protein Brahma, and active histone marks, while poor in repressive ones; (iv) yellow chromatin, in which genes are also expressed, is also rich in RNA polII and poor in repressive histone marks, but enriched in the active mark H3K36me3; and (v) black chromatin, the most prevalent, covering almost 50% of the genome and containing silent or weakly expressed genes, is devoid of active histone marks and enriched in proteins involved in chromatin condensation or heterochromatin assembly (i.e., histone H1, D1, IAL, SUUR). This classification was used to follow chromatin remodeling during neural development [100]. It emerged that genes that will be activated during neuronal differentiation belong to black chromatin in neural stem cells, are silent, and in a novel TrxG-repressive state. Conversely, in neurons, genes that are essential in neural stem cells are repressed by HP1, and not by PcG complexes, which rather regulate lineage-specific factors. This study has not only highlighted the importance of black-type and HP1 chromatins during development but has also moderated the predominant role given to PcG complexes in gene silencing. Another study characterized nine different chromatin types by following 18 histone modifications in two *Drosophila* cell lines [99]. By combining these data with genomic data, it further allowed for a fine description of chromatin signatures of functional elements.

High-resolution imaging of *D. melanogaster* tissues has also been essential to show the formation of higher-order chromatin structures [101,102,103]. PcG proteins form discrete foci called Polycomb bodies, where several repressed PREs were co-localized thanks to chromatin looping [102]. Elegant studies have shown that in *D. melanogaster* embryos, PREs of silenced genes co-localize in Polycomb bodies whereas those of active genes stay outside these foci of Polycomb proteins [101]. The analysis of chromosomal contacts using a modification of chromosome conformation capture called Hi-C revealed that intra-chromosomal repressive chromatin domains (corresponding to blue, black, and green chromatin) cluster together. In contrast, active domains (corresponding to red and yellow chromatin) are more likely to form inter-chromosomal contacts with other active domains but not with inactive ones [104]. The domains within which looping interactions occur were further called TADs. The formation of such TADs was recently analyzed using genetic manipulations and Hi-C [105]. It was thus shown that deletion of PREs using CRISPR-Cas9 genome editing prevents the formation of repressive chromatin loops and interferes with the transmission of PcG silencing during development. Moreover, the disruption of PRE regulation by PRC1 depletion induces chromatin decompaction before ectopic target gene expression [106]. These two studies show that the primary function of PcG complexes is to compact chromatin in a heritable way, thus preventing later gene activation. Interestingly, by artificially creating PRE epialleles, it was shown that 3D chromatin interactions between PREs and the level of H3K27me3 they bear underlie transgenerational inheritance and plasticity of their epigenetic state [107].

## 7. *Drosophila* Epigenetics and the Environment

How organisms cope with fluctuating environments and maintain a robust phenotype or, on the contrary, optimize their phenotype to live in these more or less hostile environments is a major ongoing topic.

In *D. melanogaster*, epigenetic mechanisms are well known to respond to the environment, and notably to temperature variations. Using transgenes bearing a *white* reporter, it was shown that PEV is increased at a low temperature, whereas Polycomb silencing is increased at a high temperature [108]. Accordingly, temperature modulates the motion of chromatin domains, Polycomb bodies, and the exchange of Polycomb molecules in Polycomb bodies [103]. Furthermore, transgenerational epigenetic inheritance of PRE epialleles was shown to be modulated by temperature and the responsive windows were identified during gametogenesis and embryogenesis [107]. Thus, many genomic regions are likely to be affected by temperature via chromatin-based mechanisms with potential phenotypic effects. This suggests that these mechanisms might underlie phenotypic plasticity, the ability of a given genotype to produce distinct phenotypes in response to different environmental conditions [109]. In *D. melanogaster* females, the pigmentation of the posterior abdomen is very sensitive to temperature, with low temperature increasing melanisation of the cuticle [110]. This is caused by a strong increase in the expression of *tan* encoding an enzyme involved in the formation of cuticular pigments [111]. This high expression correlates with an increase in H3K4me3 on the *tan* promoter. The *TrxG* gene *Trithorax* was shown to be required for the deposition of this mark and the high *tan* expression observed at low temperature. The temperature also has an effect on the activity of piRNA clusters. Indeed, a high temperature (29 °C) is able to convert an inactive piRNA cluster into an active one that produces piRNAs [112]. This conversion is then stably maintained as the converted piRNA cluster remains active in the following generations even when grown at 25 °C.

Temperature sensitivity of chromatin regulation has implications in the local adaptation of natural populations. Indeed, when populations migrate to a new environment with a distinct temperature regime, they suffer a different selection pressure. By comparing *D. melanogaster* populations from tropical (ancestral) and temperate environments, it was possible to detect selection in certain chromatin regulators or their targets (for example, PREs) [113,114,115]. Furthermore, experimental analyses of the tropical and temperate alleles have shown that they differ functionally and are likely involved in adaptation to different temperatures [115,116].

Other environmental factors were shown to affect chromatin regulation. For example, varying the level of proteins and carbohydrates in food alters the expression of many epigenetic factors (chromatin binding, histone regulators, histone modifiers, etc.) with correlated modifications in the expression of genes involved in immunity, neurotransmission, neurodevelopment, oxidative stress, and metabolism [117]. Remarkably, these changes in expression persist for two generations even if flies are grown on a standard diet.

In an experimental setup, *D. melanogaster* larvae were subjected to a toxic challenge while expressing a gene of resistance to this toxic product in spatially restricted regions using a Gal4 driver. This allowed researchers to demonstrate that development is sufficiently plastic to adapt to such challenges by increasing the number of cells expressing the resistance gene [118]. Interestingly, this rapid adaptation occurs through changes in chromatin regulation and notably through the reduced expression of *PcG* genes. Remarkably, some changes in phenotypes were inherited across multiple generations grown in the absence of the poison.

These few examples show that epigenetic mechanisms often mediate the short-term adaptive response to environmental changes and illustrate the predominant role of the PcG and TrxG genes. Interestingly, the antagonism between these two gene families extends to life history traits and resistance to environmental stress. Indeed, heterozygous mutants for the PcG genes *E(Z)* or *esc* are long lived and more resistant to oxidative stress and starvation, effects that are suppressed by a mutation in TrxG *trithorax* [119]. Strikingly, in some cases, the epigenetic state was shown to be transmitted through generations in the absence of the environmental trigger that induced them initially. This highlights the important role that epigenetics plays in evolution.

## 8. Conclusions

Many processes of chromatin regulation discovered in *D. melanogaster* are conserved in other insects such as silk worms, honeybees, and ants [120,121,122,123]. More remarkably, they are also widely conserved in metazoans and are involved in development and cancer in vertebrates [124]. The major role of epigenetic mechanisms in response to the environment extends to plants where homologues of the Polycomb complex PRC2 play an essential role in vernalization [125,126]. The role of piRNA and heterochromatin modifications in the control of transposons is also extremely conserved in mammals even if in this case it also involves DNA methylation, which is almost absent in *D. melanogaster* [127]. Thus, *D. melanogaster* has played and is still playing a major role in the study of epigenetic mechanisms, although this was entirely unexpected when it began to be used in laboratories more than a hundred years ago, or when PEV or Polycomb phenotypes started to be studied, illustrating the importance of basic research on model organisms.

## Data Availability

Not applicable.
